# Relicts from Glacial Times: The Ground Beetle *Pterostichus adstrictus* Eschscholtz, 1823 (Coleoptera: Carabidae) in the Austrian Alps

**DOI:** 10.3390/insects12010084

**Published:** 2021-01-19

**Authors:** Wolfgang Paill, Stephan Koblmüller, Thomas Friess, Barbara-Amina Gereben-Krenn, Christian Mairhuber, Michael J. Raupach, Lukas Zangl

**Affiliations:** 1 Universalmuseum Joanneum, Studienzentrum Naturkunde, Weinzöttlstraße 16, 8045 Graz, Austria; lukas.zangl@uni-graz.at; 2 Institute of Biology, University of Graz, Universitätsplatz 2, 8010 Graz, Austria; 3 Ökoteam—Institute for Animal Ecology and Landscape Planning, Bergmanngasse 22, 8010 Graz, Austria; friess@oekoteam.at; 4 Unit Integrative Zoology, Department of Evolutionary Biology, University of Vienna, Althanstraße 14, 1090 Wien, Austria; barbara-amina.gereben@univie.ac.at; 5 Amt der Steiermärkischen Landesregierung, Abteilung 16, Baubezirksleitung Steirischer Zentralraum—Naturschutz, Bahnhofgürtel 77, 8020 Graz, Austria; christian.mairhuber@stmk.gv.at; 6 Zoologische Staatssammlung München (SNSB-ZSM), Sektion Hemiptera, Münchhausenstraße 21, 81247 München, Germany; raupach@snsb.de

**Keywords:** biogeography, *Bothriopterus*, DNA barcoding, cytochrome *c* oxidase subunit 1 (COI), nunatak hypothesis

## Abstract

**Simple Summary:**

The extant distribution of many plants and animals is the result of the dynamics of the last ice ages with their recurrent advances and retreats of the northern ice sheet and the glaciers in the mountains. The arctic-alpine distribution is a special case where a species occurs in the subarctic/arctic regions and locally restricted in the alpine mountain regions of central or southeastern Europe. Among the ground beetles, several species display this type of distribution, one of which is *Pterostichus adstrictus*. In Europe, this ground beetle has been thought to have its southernmost occurrences in Wales and southern Scandinavia. In this study, we provide the first reliable record of *P. adstrictus* from the Austrian Alps based on morphological determination and comparison to other closely related species as well as molecular genetic data. Furthermore, the seasonal occurrence as well as empirical habitat preferences of *P. adstrictus* in the Austrian Alps are described.

**Abstract:**

The last ice age considerably influenced distribution patterns of extant species of plants and animals, with some of them now inhabiting disjunct areas in the subarctic/arctic and alpine regions. This arctic-alpine distribution is characteristic for many cold-adapted species with a limited dispersal ability and can be found in many invertebrate taxa, including ground beetles. The ground beetle *Pterostichus adstrictus* Eschscholtz, 1823 of the subgenus *Bothriopterus* was previously known to have a holarctic-circumpolar distribution, in Europe reaching its southern borders in Wales and southern Scandinavia. Here, we report the first findings of this species from the Austrian Ötztal Alps, representing also the southernmost edge of its currently known distribution, confirmed by the comparison of morphological characters to other *Bothriopterus* species and DNA barcoding data. Molecular data revealed a separation of the Austrian and Finish specimens with limited to no gene flow at all. Furthermore, we present the first data on habitat preference and seasonality of *P. adstrictus* in the Austrian Alps.

## 1. Introduction

With their recurrent periods of glacial advances and retreats, the Pleistocene glacial cycles are considered one of the most important drivers of current distributional patterns and patterns of genetic diversity in animals and plants [1,2,3]. This particularly applies to arctic-alpine taxa, which are characterized by a disjunct occurrence in two geographically distant areas, the subarctic to arctic plains and hills, and the high mountain ranges of the Alps and/or other Eurasian mountains [4]. This large-scale disjunction occurs in numerous species of plants and animals, typically taxa that are characterized by poor dispersal capacity [1,5,6,7,8].

Among the ground beetles, a notable number of arctic-alpine species is known. Furthermore, profound faunistic knowledge on this species group—particularly in the Alps [1,9,10]—makes this beetle family a promising model system for comparative phylogeographic studies, in particular with respect to effects of Quaternary climatic changes on current species distribution and patterns of genetic diversity.

Arctic-alpine ground beetles are generally widespread across the Tundra. In Central Europe, some of the species are quite common, inhabiting relatively large regions, such as *Nebria gyllenhali* (Schönherr, 1806), which lives in riparian habitats along mountain brooks [11,12,13]. Other species, however, have distributions split up into a few small and separated relict populations in the Alps or Carpathians, such as *Patrobus septentrionis septentrionis* Dejean, 1828 [12,14], which lives above the timberline.

In the present study we show that *Pterostichus adstrictus* Eschscholtz, 1823, is another example of this particular type of arctic-alpine distribution. This species has a holarctic-circumpolar distribution, ranging from Northern Europe to Eastern Siberia, North Korea and Japan [15,16], but is also widespread across Alaska and Canada [17]. In North America it is frequently encountered as one of the most common ground beetle species [18,19,20,21]. In Europe, however, the southernmost occurrences were documented from Northern Ireland, Wales and southern Scandinavia [22,23,24] and, thus far, there have been no records from the Alps. Interestingly, the first molecular study based on DNA barcodes revealed haplotype-sharing of specimens of *P. adstrictus* and *P. oblongopunctatus* (Fabricius, 1787) from populations of Finland and Germany [25]. Here, we describe the first findings of *P. adstrictus* in the Austrian Ötztal Alps confirmed by morphological and DNA barcode data. We further discuss morphological and mitochondrial DNA sequence variation across the species’ distribution and provide data on habitat preferences and seasonal dynamics of this species in the Alps.

## 2. Materials and Methods

### 2.1. Study Area and Sampling

Field observations and sampling were carried out in the Ötztal Alps, which are among the largest mountain groups within the Eastern Alps. They comprise several summits above 3500 m and are heavily glaciated in higher altitudes. The study area has a relatively dry intra-montane climate with low annual precipitation values: The annual mean precipitation was 1100 mm at the Gepatschalm (1903 m, Kaunertal, Austria) from 2009 to 2015 and 920 mm at the Weißsee (2465 m, Kaunertal, Austria) from 2006 to 2015. Consequently, it is part of the driest region of the Austrian Alps [26]. Starting in the Kaunertal and Taschachtal, where *P. adstrictus* was found during a faunistic inventory by chance, the investigations were further extended to eight other areas in the mountain group that were selected by extrapolating the observed habitat choice parameters (elevation, habitat type) of the species. Altogether, we looked for *P. adstrictus* in ten areas, nine of which are located in the North Tyrolean Central Alps (Austria) and one south of the Alpine main ridge in South Tyrol (Italy) (Table 1). Sampling of the ground beetles was done using pitfall traps and collection by hand. Throughout the field studies, empirical observational data regarding habitat parameters (altitude, geomorphology, vegetation) were recorded.

### 2.2. Species Determination and Morphological Comparison

Ground beetles were determined morphologically following [27]. In the case of *P. adstrictus*, we additionally used [28,29] and compared our material with specimens from Northern Europe and China/Yunnan. The nomenclature follows [30]. All ground beetle vouchers are deposited in the National History Museum Graz (Studienzentrum Naturkunde).

In order to characterize the Austrian *P. adstrictus* beetles based on morphological traits, we measured the following characters in 30 specimens: number of foveolate setigerous punctures on elytral interval 3, length of pronotum along its median line, length of elytron from the tip of the scutellum to the apex, width of the elytron at its broadest position and length and width of the hind wings. Male genitalia were prepared, cleared in lactic acid and embedded in Euparal. In order to compare *P. adstrictus* data with those of the only two other European species of the subgenus *Bothriopterus* (*Pterostichus oblongopunctatus* (Fabricius, 1787) and *Pterostichus quadrifoveolatus* Letzner, 1852), we also studied Austrian and Czech material of these species and incorporated literature data [31,32,33,34,35].

### 2.3. Molecular Characterization

Total genomic DNA of seven *P. adstrictus* specimens and three *P. oblongopunctatus* specimens (stored at the University of Graz) was extracted from leg muscle tissue using a DNeasy^®^ Blood & Tissue Kit (Qiagen, Hilden, Germany). A 658 bp fragment of the mitochondrial COI gene, corresponding to the typical DNA barcoding region sensu Hebert [36], was amplified using the Phusion polymerase (Thermo Fisher Scientific, Waltham, MA, USA) protocol, following the manufacturer’s instructions. The same primers were used for both PCR and cycle sequencing, namely C_LepFolF and C_LepFolR [37]. PCR products were purified with ExoSAP-IT (Thermo Fisher Scientific). The sequencing reaction followed the protocol in [38]. Sequencing products were purified with SephadexTM G-50 (Amersham Biosciences, Little Chalfont, UK) and visualized on an ABI 3130xl capillary sequencer (Applied Biosystems, Foster City, CA, USA). Double-stranded sequences were assembled and checked for mitochondrial pseudogenes (numts) by looking for the presence of internal stop codons, frameshifts as well as double peaks in chromatograms with MEGA 10.0.5 [39]. All new DNA barcodes were deposited at the Barcode of Life Data Systems (BOLD: www.boldsystems.org, ACAR001-21-ACAR010-21) [40]. Parallel to this, all new sequences were deposited in GenBank (accession numbers: MW472683-MW472692). For the analysis we also included all published DNA barcodes of the subgenus *Bothriopterus*, i.e., *P. adstrictus* (*n* = 18), *P. quadrifoveolatus* (*n* = 8) and *P. oblongopunctatus* (*n* = 26) (see Supplementary Material Table S1) ([25,41,42,43,44,45]).

Sequences were aligned using MUSCLE [46] as implemented in MEGA 10.0.5. MEGA was also used to calculate Kimura-2-parameter distances (K2P; [47]) among groups of interest. A statistical maximum parsimony haplotype network was constructed with TCS 1.21 based on default settings [48] implemented in the software package PopART v.1.7 [49] to infer evolutionary relationships and geographical distributions among the recorded haplotypes.

## 3. Results

### 3.1. Morphological Characterization of Austrian P. adstrictus in Comparison to Asian Populations

**Figure 1:**
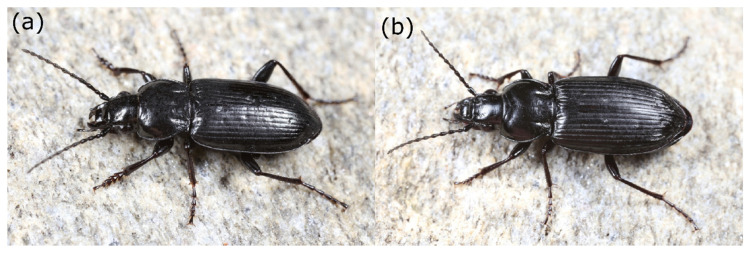
Habitus of male (**a**) and female (**b**) *Pterostichus adstrictus* from the Kaunertal (Ötztal Alps, Austria).

As one of the most striking features, the representatives of the subgenus *Bothriopterus* had distinctly foveolate setigerous punctures on the third elytral interval (Figure 1). Since there are species-specific differences with regard to this character, the number of the foveolate punctures was determined. In the alpine specimens, 50% of the elytrons had five punctures, whereas in 27% of the cases there were six and in 23% only four punctures. Notably, the number of foveolate punctures differed between the right and left elytra in more than half of the beetles examined (57%). The average number of punctures was 5.0 per elytron in the Alpine animals, while for specimens from Yunnan/China 6.6 (*n* = 20, 10 beetles) punctures per elytron were estimated. This difference between Austrian and Chinese beetles was highly significant (*t*-test, *p* < 0.0001).

The ratio of elytra length to the length of the pronotum was 2.62 ± 0.06 in the Austrian animals (Kaunertal: 2.62 ± 0.06, Taschachtal: 2.62 ± 0.06), but only 2.51 ± 0.06 in the beetles from Yunnan/China (*n* = 10). This difference between Austrian and Chinese beetles was also highly significant (*t*-test, *p* < 0.0001).

The membranous wings of *P. adstrictus* from the Ötztal Alps were well developed. They were on average 1.44 ± 0.05 times as long and 1.67 ± 0.09 times as wide as the elytra. These values hardly differed among the sexes or among the two examined populations (Kaunertal, Taschachtal).

**Figure 2:**
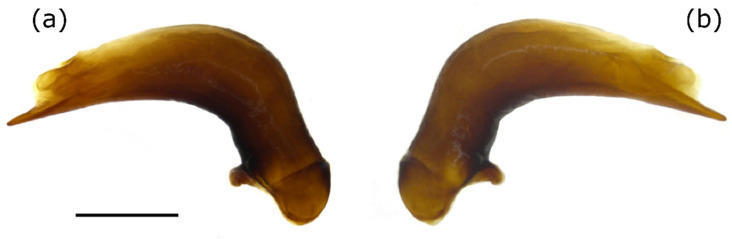
Median lobe of the aedeagus of *Pterostichus adstrictus* from the Taschachtal (Ötztal Alps, Austria), (**a**) right lateral view, (**b**) left lateral view, scale bar = 1 mm.

**Figure 3:**
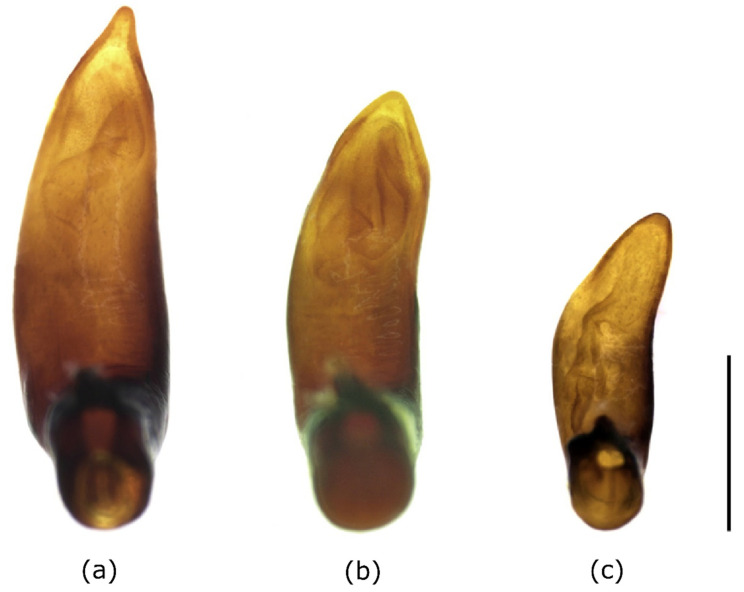
Comparison of the aedeagi of *Pterostichus adstrictus*, Kaunertal/Austria (**a**), *P. oblongopunctatus*, Rabachboden/Austria (**b**) and *P. quadrifoveolatus*, Lopenik/Czech Republic (**c**), median lobe in ventral view, scale bar = 1 mm.

The median lobe of the aedeagus of the examined specimens had a characteristic and constant external shape. This particularly applied to the tip, which in the ventral view was sharply tapered. The internal sac also showed typical structures, although sclerites were missing (Figure 2 and Figure 3a).

### 3.2. Morphological Differentiation and Determination of the European Bothriopterus

**Figure 4:**
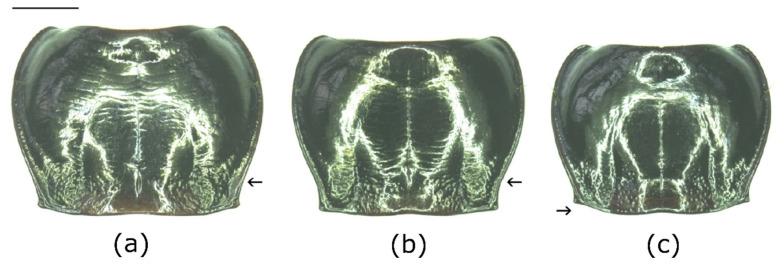
Comparison of the pronota of *Pterostichus adstrictus*, Kaunertal/Austria (**a**), *P. oblongopunctatus*, Tainach/Austria (**b**) and *P. quadrifoveolatus*, Vracov/Czech Republic (**c**), scale bar = 1 mm. The arrows indicate the widened (**a**), not widened (**b**) lateral edge of the pronotum and the angled base of the pronotum (**c**).

Within the subgenus *Bothriopterus*, *P. adstrictus* is difficult to distinguish from the other two European species, *P. oblongopunctatus* and *P. quadrifoveolatus*. Males are most reliably distinguished by comparing the outer shape of the aedeagi. In contrast to *P. adstrictus*, the tip was not tapered but rounded in the two other species (Figure 3). External morphological characters that are visible from the outside, like the shape of the pronotum (Figure 4) and the color of the tibia, can also be used. For putting together a determination table (Table 2), we considered both our newly obtained data as well as information from the literature (see above).

### 3.3. Analysis of the DNA Barcode Data

**Figure 5:**
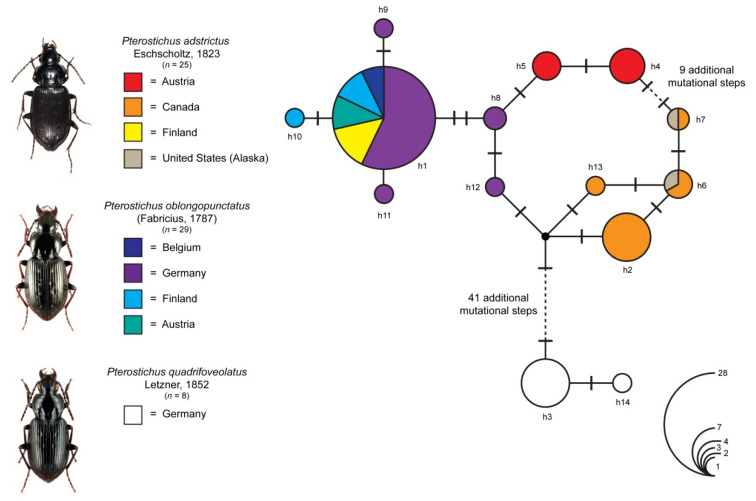
Maximum statistical parsimony network of *Pterostichus adstrictus*, *P. quadrifoveolatus* and *P. oblongopunctatus*. Default settings for connection steps were applied and gaps were treated a fifth state. Bars represent single mutational changes whereas small black dots indicate missing haplotypes. The numbers of analyzed specimens (*n*) are listed and the diameter of the circles is proportional to the number of specimens per haplotype (see open circles with numbers). Beetle images were obtained from www.eurocarabidae.de, except for *P. adstrictus* (photographer: Ditta Balke).

In total, 25 DNA barcode sequences of *P. adstrictus*, 29 sequences of *P. oblongopunctatus* and eight sequences of *P. quadrifoveolatus* were analyzed. Intraspecific K2P distances ranged from 0% to a maximum of 2.8% for *P. adstrictus*, 0% to 0.2% for *P. quadrifoveolatus* and 0% to 0.7% for *P. oblongopunctatus* (Table 3). Statistical maximum parsimony analysis revealed 14 different haplotypes (Figure 5). Haplotype-sharing was found for *P. adstrictus* specimens from Finland and *P. oblogopunctatus* beetles from Austria in a dominant haplotype (h1, *n* = 28) that was surrounded by all other haplotypes of *P. oblongopunctatus* (h8–h12), with a maximum of three additional mutational steps (h12). In contrast, all Austrian *P. adstrictus* sequences were grouped in two distinct haplotypes (h4, *n* = 4; h5 *n* = 3) that were separated from haplotype h8 (*P. oblongopunctatus* from Germany, *n* = 2) by one (h5) and two (h4) mutational steps, respectively. K2P distances between *P. adstrictus* specimens from Austria and the haplogroup comprising *P. adstrictus* from Finland and *P. oblongopunctatus* from Austria, Belgium, Finland and Germany ranged from 0.15% to 0.77% (Table 3), whereas all *P. adstrictus* specimens from North America were found in a group with four haplotypes (h2, h6, h7, h13), separated from the Austrian specimens by at least 1.69%. All specimens of *P. quadrifoveolatus* were pooled in two haplotypes (h3, h14) and, with distances >7%, distinct from all other analyzed *Pterostichus* specimens.

### 3.4. Occurrence and Habitat Use of P. adstrictus in the Ötztal Alps

*Pterostichus adstrictus* was recorded in two of the ten study areas. Despite the apparently geographically very restricted occurrence, the species’ populations in the Kaunertal and the Taschachtal are remarkably large. A total of more than 150 individuals were caught or observed. All records came from altitudes between 1875 and 2265 m above sea level.

**Figure 6:**
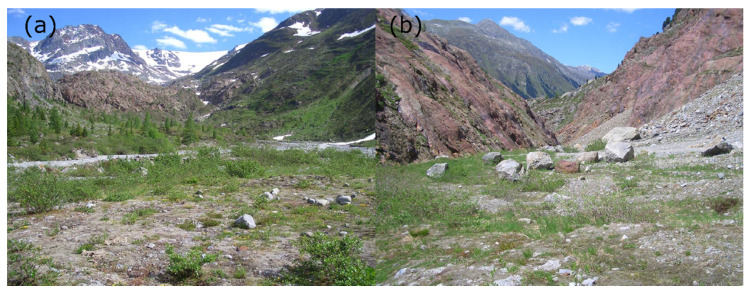
Typical habitats of *Pterostichus adstrictus* in the Kaunertal (Ötztal Alps, Austria): gravel bank (**a**), moraine (**b**).

*Pterostichus adstrictus* showed specific habitat requirements (stenotopic behaviour). We found the species to prefer pioneer stages of gravel banks along mountain brooks (Figure 6a). More dynamic and regularly flooded immediate riparian zones remain uninhabited, however. The species was also missing in the transition zones of the surrounding habitats of alpine heath, meadows and pastures. However, the more stable, rarely flooded inner and higher parts of the gravel banks were populated. Apparently, there was a special preference for areas with patchy herbaceous vegetation. Here, the beetles lived on moderately moist, sandy raw soils under larger stones.

Some specimens were also found in silicate scree slopes deposited by historical glacial activity (Figure 6b). These moraines structurally resemble gravel banks along mountain brooks, with their high proportion of sandy fractions, patchy vegetation, constant degree of soil humidity and sufficient stability of the raw soil, at least at the flattened lower slope areas, where *P. adstrictus* was captured.

Based on a biotope mapping, a potentially populated area of approximately 0.3 km^2^, 0.18 in the Kaunertal and 0.13 in the Taschachtal was assumed. An exact visualization of the areas inhabited by *P. adstrictus* was not done here in order not to endanger the populations by possible collectors.

### 3.5. Seasonality

**Figure 7:**
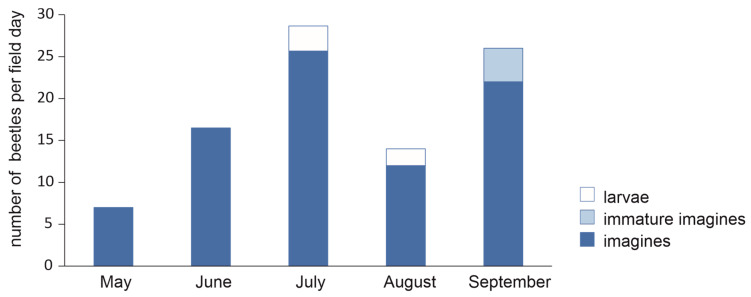
Seasonality of *Pterostichus adstrictus* in the Kaunertal, shown by caught/observed beetles in 2009 and 2010, excluding catches by pitfall traps.

The seasonal appearance of *P. adstrictus* in the Ötztal Alps starts soon after the snow melts in May. High surface activity was documented from mid-June to mid-August with a maximum during July. The second peak in September represented inactive individuals that were found in their overwintering holes under big stones. In July and August, larvae were observed, and in September, immature, newly hatched adults (Figure 7).

### 3.6. Accompanying Ground Beetle Fauna

At the local sites in the Kaunertal and the Taschachtal, *P. adstrictus* formed strikingly individual-rich populations; sometimes it was even the most common ground beetle. Among the species regularly found alongside *P. adstrictus* awee *Cicindela campestris* Linnaeus, 1758, *Nebria gyllenhali* (Schönherr, 1806), *Bembidion bualei* Jacquelin du Val, 1852, *Sinechostictus ruficornis* (Sturm, 1825), *Sinechostictus stomoides* (Dejean, 1831), *Amara erratica* (Duftschmid, 1812) and *Amara quenseli quenseli* (Schönherr, 1806). Syntopy with other larger *Pterostichini* such as *Pterostichus jurinei* (Panzer, 1802) and *Pterostichus multipunctatus* (Dejean, 1828), both common in the Ötztal Alps, was rare. The accompanying occurrences of several other arctic-alpine and boreo-montane species or of taxa with high agreement with these types of distribution were striking. These included the above-mentioned *N. gyllenhali*, *A. erratica* and *A. quenseli quenseli* as well as *Trechus rubens* (Fabricius, 1792), *Miscodera arctica* (Paykull, 1798) and *Cymindis vaporariorum* (Linnaeus, 1758).

## 4. Discussion

### 4.1. Morphological Characterization

*Pterostichus adstrictus* belongs to the species-poor subgenus *Bothriopterus*, which has two main distribution areas, one in East Asia and one in North America [15,50,51]. In addition to *P. adstrictus*, two other *Bothriopterus* spp. are documented in Europe: *P. oblongopunctatus* and *P. quadrifoveolatus*. All three are relatively difficult to distinguish from one another, but based on characteristics of the pronotum and of male genitalia they are characterized as separate species. In scientific literature, there is no doubt concerning their taxonomic status [15,32,51,52].

Within its large distribution area, the morphology of *P. adstrictus*, and especially the prothorax shape, varies [18]. In the past, a large number of taxa have been described that are currently considered synonyms of *P. adstrictus* [15,53]. The beetles from the Ötztal Alps, in fact, showed slightly different body proportions in comparison to specimens from northeastern North America. For example, the elytra were slightly longer in relation to the pronotum in the alpine populations (mean = 2.62) than in the latter ones (mean = 2.51; [29]). The same value as in North America was found in specimens from Yunnan/China (mean = 2.51).

Furthermore, the number of foveolate punctures in the beetles from the Ötztal Alps seemed to be reduced. Although no statistical data exists about the number of this punctures from other populations of *P. adstrictus*, [18] (p. 486) gave an indication on that. He stated, that there are five or six and “very seldom 4” punctures on the third elytral interval. The specimens used for comparison from Yunnan/China also had a significantly higher average number of points than those from the Ötztal Alps. However, it is uncertain, whether this difference has taxonomic significance. It might well be that this difference in the number of foveolate punctures was due to specific environmental conditions, as has been previously shown for *P. oblongopunctatus*, with the number of pits on the elytra being related to soil moisture [54,55].

On the other hand, the male genitalia seemed to be highly conserved across the whole distribution area. When comparing animals from the Ötztal Alps with that from Yunnan/China, no differences in the external shape of the aedeagi were found. Also, male genitalia of North American specimens seemed to be similar, as indicated by an illustration by [18], to which [29] refers to.

### 4.2. DNA Barcode Analysis and Origin of Alpine Populations

The arctic-alpine disjunction observed in numerous cold-adapted species is usually caused by Pleistocene climatic oscillations. During glacial maxima, the Alps were nearly entirely covered by ice [56,57]. Consequently, species survival was mostly restricted to larger unglaciated areas at the Alpine periphery, particularly in the southwestern, southern and eastern parts. From there, numerous species managed to repopulate former devastated areas [58,59]. In a few cases, confirmed based on genetic data [60,61], a scenario that seems to also apply to the distribution pattern of *Pterostichus adstrictus*. This hypothesis is also supported by the co-occurrence long-term survival in inner alpine regions on ice-free mountaintops (nunataks) has been recently of *Miscodera arctica*, another species with a highly disjunct arctic-alpine distribution in the Kaunertal.

The analysis of the molecular data confirmed a clear differentiation of the European specimens of *Pterostichus adstrictus* from those of North America, indicating a lack of gene flow in the recent past. This also becomes evident in terms of genetic distances, with values ranging from 1.69% up to 2.17%. The most prevalent haplotype of Northern European *P. adstrictus* was shared with *P. oblongopunctatus* from Central and Northern Europe [25,42], whereas the Austrian *P. adstrictus* formed a separate but closely related clade with a minimum distance of 0.15% to a haplotype of *Pterostichus oblongopunctatus* (Figure 5). Application of the widely used “conventional” phylogenetic COI divergence rate of 2.3% per MY [62] would imply that the alpine and artic populations diverged in the Middle Pleistocene, ~300 KYA, but well before the last glacial maximum (~18–25 KYA). Indeed, this “conventional” divergence rate for the COI is not universal, with quite some variation, especially among higher taxa [63]. In addition, there is increasing empirical evidence for a time dependency of the molecular clock, with phylogenetic substitution rates considerably overestimating recent events on the population level [64,65,66]. Hence, it might well be that the alpine and arctic populations split much more recently.

The lack of mitochondrial divergence between *P. oblongopunctatus* and northern *P. adstrictus*, despite the clear differences in morphology [31,33,67], indicates either recent divergence or, more likely, a recent mitochondrial introgression event. However, the putative direction of this suggested phenomenon remains unclear with the data available. There is increasing evidence from many different animal species that range-wide (or across large parts of a species’ distribution) mitochondrial replacement is more common than previously thought [68,69,70,71] and hybridization/introgression has been repeatedly reported in carabid beetles [72,73,74], including the genus *Pterostichus* [43,75]. Unfortunately, here, mitochondrial data alone are insufficient to clarify which processes underlie the observed haplotype sharing between *P. oblongopunctatus* and northern *P. adstrictus*. In addition, no hybrids in Scandinavia are known so far. To conclusively disentangle the effects of recent divergence and potential introgression and infer robust population divergence times in *P. adstrictus*, comprehensive nuclear multilocus data of more specimens from different localities are needed.

### 4.3. Faunistic Interpretation and Historic Data

The discovery of *P. adstrictus* can be considered a surprise. Even though the species clearly has a very restricted distribution in the Alps, it is very likely that it spans a larger area than just the two areas where we found it. At the two sites where we found *P. adstrictus*, this conspicuously large beetle was very abundant. In general, the Ötztal Alps are well studied in terms of coleopterology, both in historical [76,77,78,79,80,81,82] and also more recent times [83,84,85,86,87,88], with a particular focus on Obergurgl at the eastern border of the Ötztal Alps [89,90,91]. Yet, none of these previous studies reported a potential presence of *P. adstrictus* in the Ötztal Alps.

However, literature suggests that our findings of *P. adstrictus* may indeed not be the first for Austria. In fact, specimens reported as *P. oblongopunctatus* by [80] in the Kaunertal from elevations of 1930 m above sea level could actually refer to *P. adstrictus*. The two species are easy to confuse and, in the Alps, the former only exceptionally exceeds 1500 m above sea level [92,93,94]. Additionally, there are unconfirmed reports about a population of *P. adstrictus* in the Hohe Tauern. This large mountain group is the highest part of the Eastern Alps, situated about 70 km east of the Ötztal Alps. Finally, ref [95] reported *Pterostichus borealis* Zetterstedt, [96] *Platysma oblongopunctata* var. *borealis* Zetterstedt and [97]—apparently just following [95]—reported *Pterostichus vitreus* Dejean, each from Austria’s biggest glacier Pasterze. However, these reports were previously not considered in the literature [98,99,100] or regarded as untrustworthy [101,102]. A more recent search for *P. adstrictus* in the area of the Pasterze glacier was unsuccessful and it still remains unclear whether historical collection material can bring certainty about a further occurrence of *P. adstrictus* in the Alps.

### 4.4. Habitat Preference and Physiological Adaptations

The relict populations of *P. adstrictus* in the Alps are restricted to the alpine region above the timberline and show stenotopic habitat use. In contrast, the circumpolar populations of the species are more eurytopic, inhabiting a variety of habitats from the northern coniferous forest to the subarctic region [103].

Consistent with the observations from the Ötztal Alps, *P. adstrictus* in Northern Europe prefers forest-free, open (grassland) habitats on gravel soils (moraine) with sparse and patchy vegetation [22,103]. However, the species additionally uses open forests and forest edges in Scandinavia [103], apparently prefers moors and moist heath locations in Ireland and England [23,24], regularly occurs in cultivated arable land in northern Norway [22,67] and in moderately moist to dry meadows in Iceland [104]. Contrasting biotope preferences from different parts of the area are also known from Siberia [105]. Thus, *P. adstrictus* colonizes forests in the extreme continental sector, while in the eastern transitional sector, open habitats are preferred.

In North America, *P. adstrictus* can be found from coastal regions to high altitudes in the mountains and accordingly inhabits an even broader range of biotope types such as forests, hedges, meadows, fields, coastal tundra and even gravel pits, gardens and roadsides [106]. There, the species is classified as a eurytopic forest dweller. It prefers coniferous forests, frequently living on its edges or in clearings and regularly benefitting from forest fires and wind breaks [107,108,109,110].

The common denominator characterizing all of the *P. adstrictus* habitats seems to be spatiotemporal dynamics and instability. For example, semiopen forests require periodic turnover processes, as do locations characterized by glaciers or mountain streams. Populating habitats with temporal and spatial instability would require certain mobility. Indeed, spontaneous flight observations [111], catches from flight interception traps [112] and findings from artificial light sources [106] have been reported for *P. adstrictus*. Records from drifted detritus [106] and findings from a young volcanic island [104] also indicate the species’ ability to fly. This should also apply to the beetles found in the Ötztal Alps. Indeed, the membranous wings of the examined beetles were well developed. However, the measured values did not match those of regular flyers such as *Harpalus rufipes* (De Geer, 1774) or *Bradycellus harpalinus* (Audinet-Serville, 1821) [113]. In addition to the size of the hind wings, another anatomical feature, namely the presence of flight musculature, should be used to assess the individual’s ability to fly. Although this has not been investigated in the present study, it can be assumed that populations with such wing shapes do produce individuals with flight ability.

The findings on the seasonal activity of *P. adstrictus* in the Ötztal Alps agree with data for Scandinavian populations [114,115]. The species is a spring breeder with summer larvae, although findings of newly hatched beetles in the early summer [53,115] and of gravid females in autumn [18,106] indicate that hibernation in the larval or even pupal stage might exceptionally occur. The species therefore has a flexible life cycle, contrary to other ground beetle species with summer larvae that are “true summer breeders” with adult hibernation only. Gonad maturation appears far less linked to the change from short to long day conditions than it does for the closely related temperate species, *P. oblongopunctatus* and *P. quadrifoveolatus* [116]. Furthermore, the ability of *P. adstrictus* to develop quicker and more successfully in a wider temperature range than found in temperate species seems to be an adaptation to the special climate of the (sub)artic region.

### 4.5. Accompanying Ground Beetle Fauna

The fauna co-occurring with *P. adstrictus* contains a considerable number of arctic-alpine, boreo-montane or similarly ranging species. The finding of *Miscodera arctica* in the Kaunertal is particularly noteworthy. This species populates a huge circumpolar, arctic-alpine range, which is quite similar in extent to that of *P. adstrictus*. The small alpine area consists of some disjunct populations, from Graubünden (Berniner Alps to Ortler Alps), via Vorarlberg (Verwallgruppe) to South, North and East Tyrol (Ötztal Alps, Stubaier Alps, Sarntaler Alps, Dolomites, Villgratner Berge, Kreuzeckgruppe) [79,93,117,118,119]. *Miscodera arctica* has now been documented for the first time from the part of the Ötztal Alps north of the Alpine main ridge. *Cicindela campestris*, *Amara erratica* and *Cymindis vaporariorum*, three drought-tolerant species [27,120], were found together with *P. adstrictus*, indicating that also *P. adstrictus* might prefer rather dry habitats in the Alps.

## 5. Conclusions

*Pterostichus adstrictus* is known as a cold-adapted ground beetle with a circumpolar distribution. In the present study we have shown, based on both morphological and DNA barcoding data, that the species is not restricted to the arctic/subarctic region. Pleistocene relict populations are also present in the Alps, where this species appeared to be quite abundant in suitable habitats. We found the species only at two sites in the Ötztal Alps, but it is very likely that it is also present in other alpine regions, though probably, not particularly common. Haplotype sharing between northern *P. adstrictus* populations and *P. oblongopunctatus* indicates recent divergence and/or mitochondrial introgression in regions of current or past sympatry and calls for follow-up studies. In order to clarify the phylogenetic relationships and extent of potential interspecific geneflow in the subgenus *Bothriopterus* and the temporal patterns of intraspecific divergence within *P. adstrictus,* nuclear multilocus data should be applied.

## Figures and Tables

**Table 1:** Sampling localities and dates. The table lists details about the origin of the samples as well as the dates and ranges of altitudes that have been sampled by different collectors. Geographic coordinates are not presented in order not to endanger the populations by possible collectors.

Sampling Date	Origin	Collector	Altitude
11.08.2009	AUT, North Tyrol, Fissladbachtal	Christian Mairhuber	1990–2230 m
16.07.2009	AUT, North Tyrol, Griestal near Mandarfen	Wolfgang Paill	1840–2000 m
17.06.2009, 18.06.2009, 13.07.2009, 14.07.2009, 15.07.2009, 11.08.2009, 25.08.2009, 01.09.2009, 17.06.–14.07.2009, 10.05.2010, 22.09.2010, 16.07.2016, 13.08.2016	AUT, North Tyrol, Kaunertal	Thomas Frieß, Barbara-Amina Gereben-Krenn, Christian Mairhuber, Wolfgang Paill	1870–2270 m
11.08.2009	AUT, North Tyrol, Kaiserbergtal	Wolfgang Paill	2160–2520 m
11.08.2009, 09.06.2010, 13.07.2010, 21.07.2010, 18.08.2007, 19.08.2010, 21.09.2010, 09.06.-13.07.2010, 13.07.-19.08.2010, 19.08.-21.09.2010	AUT, North Tyrol, Platzertal	Thomas Frieß, Wolfgang Paill	2100–2520 m
12.08.2009	AUT, North Tyrol, Radurschlbachtal	Thomas Frieß, Christian Mairhuber	2210–2280 m
12.08.2009	AUT, North Tyrol, Rifflbachtal	Wolfgang Paill	2230–2410 m
13.08.2009	AUT, North Tyrol, Rofental	Christian Mairhuber, Wolfgang Paill	2340–2390 m
18.06.2009, 15.07.2009, 26.08.2009	AUT, North Tyrol, Taschachtal	Christian Mairhuber, Wolfgang Paill	1970–2110 m
12.08.2009	ITA, South Tyrol, Langtauferertal	Wolfgang Paill	1890–2020 m

**Table 2:** Synoptic key for the three European *Pterostichus* species of the subgenus *Bothriopterus*. The determination characters are compiled from own new data and the literature [27,28,29].

	*P. adstrictus*	*P. oblongopunctatus*	*P. quadrifoveolatus*
Number of the foveolate punctures on 3rd elytral interval	mostly 5 or 6 (4–8)	mostly 4 (2–9)	mostly 3 (2–4)
Base of the pronotum from inner impression to posterior angle	almost straight or moderately angled forwards laterally	almost straight	angled forwards laterally
Side border of the pronotum	moderately wide, widened towards posterior angle	narrow, not widened towards posterior angle	moderately wide, not widened towards posterior angle
Vertex (head behind the eyes)	without punctures	without punctures	with punctures
Color of the elytra	black, with bronzed luster, in females dull	black, with bronzed to greenish luster	black, with bronzed luster
Color of the tibiae	blackish, almost as dark as femora	reddish-brownish, distinctly paler than femora	blackish, almost as dark as femora
Color of the palpi	Blackish	reddish-brownish	blackish
1st antennal segment	as long as 3rd segment	as long as 3rd segment	clearly shorter than 3rd segment
Shape of the aedeagus in lateral view	less evenly arcuate, apex long	more evenly arcuate, apex short	more evenly arcuate, apex long
Shape of the aedeagus in ventral view	aedeagus almost straight, apex acuminate	aedeagus weakly bent, apex almost obtuse	aedeagus clearly bent, apex obtuse
Size of the aedeagus in lateral view	big (about 3 mm)	big (about 3 mm)	small (about 2 mm)
Size of the membranous wings	relative long (ratio wing to elytra >1.3)	relative short (ratio wing to elytra <1.3)	relative long (ratio wing to elytra >1.3)
Total body length (data from literature)	10.4–13 mm	9.5–13.0 mm (mean: 11.4)	8.5–11.9 mm (mean: 10.3)

**Table 3:** Pairwise K2P-distances within and between distinct groups in the haplotype network (Figure 5).

	*P. adstrictus* (Austria)	*P. adstrictus* (North America)	*P. adstrictus* (Finland)/ *P. oblongopunctatus* (Austria/Belgium/ Finland/Germany)	*P. quadrifoveolatus* (Germany)
***P. adstrictus*** **(Austria)**	0–0.0015			
***P. adstrictus*** **(North America)**	0.0169–0.0217	0–0.003		
***P. adstrictus*** **(Finland)/** ***P. oblongopunctatus*** **(** **Austria/Belgium/** **Finland/Germany)**	0.0015–0.0077	0.0201–0.028	0–0.0061	
***P. quadrifoveolatus*** **(Germany)**	0.0720–0.0753	0.0702–0.0737	0.0702–0.0707	0–0.0015

## Data Availability

The morphological data presented in this study are available on request from the corresponding author. The DNA sequence data generated/used in this study are available on GenBank under the accession numbers listed in Supplementary Table S1. Newly generated genetic data are also available on BOLD via the Process IDs ACAR001-21 to ACAR010-21.

## Supplementary Materials

The following are available online at https://www.mdpi.com/2075-4450/12/1/84/s1, Table S1: List of sequences used in the present study. The table contains the species, their origin, their GenBank or BOLD ID and the respective reference of all sequences generated in the present study or downloaded from online repositories analyzed in the present study.
