# Supplementary material for: Relicts from Glacial Times: The Ground Beetle Pterostichus adstrictus Eschscholtz, 1823 (Coleoptera: Carabidae) in the Austrian Alps
Source: Insects. 2021 Jan 19;12(1):84. doi: 10.3390/insects12010084 (PMC7835791; doi:10.3390/insects12010084)
Supplement: Supplementary file 1 [file insects-12-00084-s001.pdf]

Supplementary Table S1: List of all analyzed specimens including taxonomic classification, GenBank accession numbers, geographic origin and references.

Taxon	GenBank accession number	Geographic region	Reference
Pterostichus adstrictus Eschscholtz, 1823	MW472685	Austria	This study
	MW472686	Austria	This study
	MW472687	Austria	This study
	MW472688	Austria	This study
	MW472689	Austria	This study
	MW472690	Austria	This study
	MW472692	Austria	This study
	KJ202848	Canada	Woodcock et al. unpublished
	KJ202876	Canada	Woodcock et al. unpublished
	KM843179	Canada	Barcoding Canada Data Release
	KM843300	Canada	Barcoding Canada Data Release
	KM844086	Canada	Barcoding Canada Data Release
	KM845037	Canada	Barcoding Canada Data Release
	KM846947	Canada	Barcoding Canada Data Release
	KM847717	Canada	Barcoding Canada Data Release
	KM848678	Canada	Barcoding Canada Data Release
	KM849995	Canada	Barcoding Canada Data Release
	KM850185	Canada	Barcoding Canada Data Release
	KJ962758	Finland	Pentinsaari et al. 2014
	KJ962831	Finland	Pentinsaari et al. 2014
	KJ964662	Finland	Pentinsaari et al. 2014
	KJ965468	Finland	Pentinsaari et al. 2014
	KJ967289	Finland	Pentinsaari et al. 2014
	KU876027	USA (Alaska)	Sikes et al. 2017
	KU876028	USA (Alaska)	Sikes et al. 2017
Pterostichus oblongopunctatus (Fabricius, 1787)	MW472683	Austria	This study
	MW472684	Austria	This study
	MW472691	Austria	This study
	HQ953457	Belgium	iBOL Data Release
	KM451518	Belgium	Hendrich et al. 2015
	KJ962025	Finland	Pentinsaari et al. 2014
	KJ963063	Finland	Pentinsaari et al. 2014
	KJ964909	Finland	Pentinsaari et al. 2014
	KM441133	Germany	Hendrich et al. 2015
	KM442864	Germany	Hendrich et al. 2015
	KM443273	Germany	Hendrich et al. 2015
	KM443337	Germany	Hendrich et al. 2015
	KM445411	Germany	Hendrich et al. 2015
	KM445860	Germany	Hendrich et al. 2015
	KM446897	Germany	Hendrich et al. 2015
	GU347323	Germany	Raupach et al. 2010
	GU347324	Germany	Raupach et al. 2010
	GU347325	Germany	Raupach et al. 2010
	GU347326	Germany	Raupach et al. 2010
	GU347327	Germany	Raupach et al. 2010
	JF889460	Germany	iBOL Data Release

	MN454552	Germany	Raupach et al. 2020
	MN454563	Germany	Raupach et al. 2020
	MN454567	Germany	Raupach et al. 2020
	MN454578	Germany	Raupach et al. 2020
	MN454599	Germany	Raupach et al. 2020
	MN454681	Germany	Raupach et al. 2020
	MN454705	Germany	Raupach et al. 2020
	MN454719	Germany	Raupach et al. 2020
Pterostichus quadrifoveolatus Letzner, 1852	KM444608	Germany	Hendrich et al. 2015
	KM472288	Germany	Hendrich et al. 2015
	MN454537	Germany	Raupach et al. 2020
	MN454540	Germany	Raupach et al. 2020
	MN454587	Germany	Raupach et al. 2020
	MN454675	Germany	Raupach et al. 2020
	MN454693	Germany	Raupach et al. 2020
	MN454697	Germany	Raupach et al. 2020

References

1. Hendrich L, Morinière J, Haszprunar G, Hebert PDN, Hausmann A, Köhler F, Balke M (2015): A comprehensive DNA barcode database for Central European beetles with a focus on Germany: Adding more than 3,500 identified species to BOLD. Molecular Ecology Resources 15: 795-818.
2. Pentinsaari M, Hebert PDN, Mutanen M (2014): Barcoding beetles: a regional survey of 1872 species reveals high identification success and unusually deep interspecific divergences. PLOS One 9: e108651.
3. Raupach MJ, Astrin JJ, Hannig K, Peters MK, Stoeckle MY, Wägele JW (2010): Molecular species identifications of Central European ground beetles (Coleoptera: Carabidae) using nuclear rDNA expansion segments and DNA barcodes. Frontiers in Zoology 7: 26.
4. Raupach MJ, Hannig K, Morinière J, Hendrich L (2020): A DNA barcode library for ground beetles of Germany: the genus *Pterostichus* Bonelli, 1810 and allied taxa (Insecta, Coleoptera, Carabidae). ZooKeys 980: 93-117.
5. Sikes DS, Bowser M, Morton JM, Bickford C, Meierotto S, Hildebrandt K (2017): Building a DNA barcode library of Alaska`s non-marine arthropods. Genome 60: 248-259.
6. Woodcock TS, Boyle E, Roughley RE, Kevan PG, Labbee RN, Smith ABT, Goulet H, Steinke D, Adamowicz SJ (2013): The diversity and biogeography of the Coleoptera of Churchill: insights from DNA barcoding. BMC Ecology 13: 40.
